# TRPM6 N-Terminal CaM- and S100A1-Binding Domains

**DOI:** 10.3390/ijms20184430

**Published:** 2019-09-09

**Authors:** Monika Zouharova, Petr Herman, Kateřina Hofbauerová, Jiri Vondrasek, Kristyna Bousova

**Affiliations:** 1 Institute of Organic Chemistry and Biochemistry, Czech Academy of Sciences, Flemingovo namesti 2, 160 00 Prague 6, Czech Republic; 2 Second Faculty of Medicine, Charles University, V Uvalu 84, 150 06 Prague 5, Czech Republic; 3 Faculty of Mathematics and Physics, Charles University, Ke Karlovu 5, 121 16 Prague 2, Czech Republic (P.H.) (K.H.); 4 Institute of Microbiology of the Czech Academy of Sciences, Videnska 1083, 142 20 Prague 4, Czech Republic

**Keywords:** TRPM6, calmodulin binding motif, binding domain, CaM and S100A1, fluorescence anisotropy, molecular modelling

## Abstract

Transient receptor potential (TRPs) channels are crucial downstream targets of calcium signalling cascades. They can be modulated either by calcium itself and/or by calcium-binding proteins (CBPs). Intracellular messengers usually interact with binding domains present at the most variable TRP regions—N- and C-cytoplasmic termini. Calmodulin (CaM) is a calcium-dependent cytosolic protein serving as a modulator of most transmembrane receptors. Although CaM-binding domains are widespread within intracellular parts of TRPs, no such binding domain has been characterised at the TRP melastatin member—the transient receptor potential melastatin 6 (TRPM6) channel. Another CBP, the S100 calcium-binding protein A1 (S100A1), is also known for its modulatory activities towards receptors. S100A1 commonly shares a CaM-binding domain. Here, we present the first identified CaM and S100A1 binding sites at the N-terminal of TRPM6. We have confirmed the L520-R535 N-terminal TRPM6 domain as a shared binding site for CaM and S100A1 using biophysical and molecular modelling methods. A specific domain of basic amino acid residues (R526/R531/K532/R535) present at this TRPM6 domain has been identified as crucial to maintain non-covalent interactions with the ligands. Our data unambiguously confirm that CaM and S100A1 share the same binding domain at the TRPM6 N-terminus although the ligand-binding mechanism is different.

## 1. Introduction

Transient receptor potential melastatin (TRPM) channels are an eight-member subfamily of the cation-permeable transient receptor potential (TRP) superfamily, which exhibits heterogeneous functions and expression patterns [1]. The TRPM6 channel acts as a voltage-independent divalent cation channel with a 5-fold higher permeability for Mg^2+^ than for Ca^2+^ [2]. This channel is a tissue-specific receptor predominantly expressed in the intestinal and renal epithelium, where it provides absorption and reabsorption of Mg^2+^ [2,3,4]. TRPM6 is also rarely expressed in smooth tissue muscles [5] where the calcium-binding proteins (CBPs) studied in this research are also expressed here, although in lower expression levels [6,7,8]. The mutations of TRPM6 have been associated with hypomagnesemia and secondary hypocalcemia leading to developmental delays and to affected adults may be having seizures and tetany [9,10,11]. 

The whole TRP family forms large, mostly homotetramer functional complexes [12]. It is predicted that TRPM6 forms homotetramers [13,14], though it has been found that TRPM6, with its closest homologue TRPM7, co-assembles into heterotetramers [2,15]. A TRP monomer subunit contains six α-helical transmembrane segments (S1–S6) with selective pores between S5 and S6 along with intracellular N- and C-termini [16,17,18,19,20]. TRP cytoplasmic tails substantially differ between members and contain multiple binding motifs and domains [21,22] affecting channel function [23]. The structural information on TRPM7 has recently been obtained by Cryogenic Electron Microscopy (Cryo-EM) methods [22] which has indirectly provided more information on TRPM6 function of which the structural information is not yet available. TRP modulation is affected by many intracellular and extracellular binding agents; they commonly stimulate the activity of the channel or inhibit it [24]. Most of the TRP members (primarily canonical, vaniloid, and melastatin) can be regulated by Ca^2+^ in Ca^2+^ or CaM—dependent environment [25,26,27]. CBPs like CaM and Calcium-binding protein 1 (CaBP-1) are known as calcium-dependent TRP modulators with specific binding motifs within the TRP intracellular tails [28]. Although the binding sites of CBPs have been reported in various TRPM channels, such in vitro and in vivo, data are completely lacking for TRPM6 [29,30,31,32]. The modulation of TRPM6 has been studied by electrophysiology approaches and has revealed the control of TRPM6 activation and Mg^2+^ influx by phosphatidylinositol 4, 5-bisphosphate (PIP2) [33]. The hydrolysis of PIP2 by phospholipase C in the presence of other agonists can lead to TRPM6 gating. Otherwise, TRPM6 activity is suppressed by free intracellular Mg^2+^ [14,34,35]; channel regulation by Ca^2+^ has not been proved [34,35,36].

Transient receptor potential (TRPs) channels are well-known integrators of Ca^2+^ signalling cascades exhibiting different modes of Ca^2+^-dependent modulation. The widespread Ca^2+^-dependent mechanism of TRP-channel modulation is based on the interaction with CBPs [37]. One of the most important eukaryotic CBPs is CaM. It is a monomeric protein composed of N- and C-terminus domains linked by a flexible linker [38,39,40]. Each of the domains contains two helix-loop-helix conformations known as “EF-hand” motifs. Upon Ca^2+^ binding, the helices creating EF-hands reorient, which leads to the more hydrophobic extended conformation of CaM [41]. As a result, the CaM hydrophobic patch is exposed to the CaM surface and becomes accessible for interaction with the target receptor (examples of many identified receptor hydrophobic motifs: 1–5–10, 1–8–14, or isoleucine-glutamine “IQ” motif) [42] Since CaM EF-hand domains differ in Ca^2+^-binding affinities, CaM can act as an activator or inhibitor depending on the Ca^2+^ occupancy of each EF-hand [43,44]. The other CBP modulator of membrane receptors is S100A1 [45]. This homodimer protein forms a canonical EF-hand domain at the C-terminus and a pseudo-EF-arm at the N-terminus [46]. Upon S100A1/Ca^2+^ complex formation, canonically oriented EF-hand spirals are transferred, which results in exposure of the hydrophobic patch on the S100A1 surface. This S100A1 patch causes similar changes in the target receptor-binding domain to CaM. The first described structure of the whole TRP channel in the interaction with CaM was solved by Cryo-EM on the transient receptor potential vanilloid 6 channel (TRPV6) [42]. In this case, the TRPV6 is inactivated by binding to CaM. The structure revealed six separate TRPV6 surface areas, ensuring the binding of only one CaM molecule. Despite the abundant characterized shared CaM- and S100A1-binding domains at membrane receptors [30,31,47], the only structural analysis of the S100A1-binding mechanism was solved at ryanodine receptor 1 (RyR1) [45]. The modulating function of the S100A1 protein has not yet been demonstrated at any TRP receptor.

This paper provides the characterisation of new binding domains for CaM and S100A1 present at the TRPM6 N-terminus. In order to investigate the role of specific basic amino acids at the TRPM6 domain potentially involved in CaM and S100A1 complex formations, we have designed alanine-scanning mutations of the TRPM6 domain in the specific amino acid positions. We have found that, although the CaM- and S100A-binding domains overlap, the TRPM6/CaM and TRPM6/S100A1 complex interfaces differ.

## 2. Results

### 2.1. The Identification of TRPM6np Binding Domains for CaM and S100A1

It is known that the CaM-binding domains on TRPs exhibit two important characteristics: the hydrophobic motifs (1–4/5, 1–10, 1–14, 1–5–10, 1–7–10, 1–8–14, and IQ motif) and a cluster of at least 2 positively charged amino acids [45,47,48]. The TRPs binding domains hydrophobic motifs participate in formations of first contacts with the hydrophobic patch of the CaM/ Ca^2+^ complex (the detail mechanism of CaM and S100A1 interactions is described in the Introduction). The hydrophobic contacts between the TRP binding domain and CaM or S100A1 can often induce structural changes of the TRP binding domain which help to prepare it for specific (predominantly non-covalent) interactions with the ligands [30,31].

**Figure 1:**
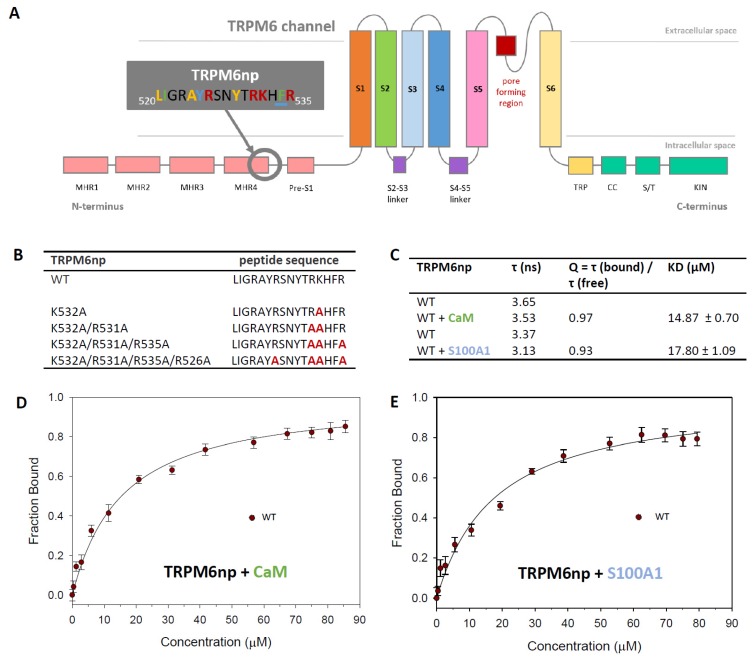
TRPM6np binding domains for CaM and S100A1: (**A**) Schematic representation of the TRPM6 channel. TRPM6 is composed of six transmembrane domains (S1–S6), a pore region between S5 and S6 with a pore helix, and intracellular N- and C-termini. The individual domains are labelled by different colours. The TRPM6 scheme (according to TRPM7 structure) presents the N-terminal tail with four melastatin homology regions (MHR 1–4) and pre-S1 domain. The TRPM6 C-terminus is composed of TRP, coiled-coil (CC), Ser/Thr (S/T), and kinase (KIN) domains. Predicted CaM and S100A1 binding domain in the grey box shows the TRPM6np sequence (L520-R535) containing several CaM recognition hydrophobic motifs (Y525-F534 (1–10 motifs) highlighted in blue, L520-A524-Y529 (1–5–10 motifs) highlighted in yellow, and I521-F534 (1–14 motifs) highlighted in green). TRPM6np basic amino acids (highlighted in red) represent a cluster of amino acids potentially involved in the interactions with CaM and S100A1. (**B**) TRPM6np amino acid sequences of wild type (WT) and mutated analogues used for the investigation of CaM and 100A1 binding site (substitutions of R/K for Figure 1A highlighted in red). The TRPM6np binding affinity to CaM and S100A1 was investigated by the steady-state fluorescence anisotropy method. This technique is based on measurement of the changing orientation of a molecule in space with respect to the time between the absorption and emission events. (**C**) The table represents a summary of the fluorescence lifetimes of fluorescein-5-isothiocyanate (FITC)-labelled TRPM6npWT (free and bound), the corresponding correction factors, and the resultant equilibrium dissociation constants of the TRPM6npWT/CaM and TRPM6npWT/S100A1 complexes obtained by steady-state fluorescence anisotropy measurements. The bound fractions of FITC-labelled TRPM6np as a function of CaM (**D**) and S100A1 (**E**) concentrations were obtained by steady-state fluorescence anisotropy measurement. The fraction bound (F_B_) of TRPM6npWT calculated according to Equation (1) was plotted against particular CaM or S100A1 concentrations, and the best fit obtained using Equation (2) gave the binding isotherms (solid lines; see the Methods section). The error bars represent the standard deviation obtained from at least five measurements.

The search for potential CaM-binding motifs at TRPM6 was performed using the Calmodulin Target Database [49]. We have identified a potential CaM-binding motif at the proximal TRPM6 N-terminus (specified as TRPM6np; see the Methods section, first caption). TRPM6np contains several hydrophobic motifs with high propensity to form interactions with CaM (Y525-F534 (1–10 motifs), L520-A524-Y529 (1–5–10 motifs), and I521-F534 (1–14 motifs)—Figure 1A). TRPM6np also involves a cluster of five basic residues: R523, R526, R531, K532, and R535. We have predicted R526, R531, K532, and R535 as essential for specific interactions with CaM. Due to a known overlap of CaM and S100A1 binding sites [30,31,45], we anticipated similar binding principles in the formation of the TRPM6np/S100A1 complex. The TRPM6npWT peptide and its mutants were prepared as described in the Methods section, Section 4.1 (Figure 1B), and they were used for in vitro and in silico experiments to confirm and characterize the predicted CaM- and S100A1-binding domains.

### 2.2. CaM- and S100A1-Binding Domains of the TRPM6 N-Termini Overlap

The binding affinity of TRPM6np to CaM and S100A1 was quantified by steady-state fluorescence anisotropy experiments. Fluorescein-5-isothiocyanate (FITC)-labelled TRPM6npWT was titrated with increasing aliquots of CaM or S100A1, and the fluorescence anisotropy value was recorded for each CaM or S100A1 addition. Due to the Ca^2+^ dependence of TRPs/CBPs interactions, all measurements were carried out in the presence of 2 mM CaCl_2_ [30]. Upon both TRPM6npWT/CaM and TRPM6npWT/S100A1 complex formation, an increase in fluorescence anisotropy resulting from decreased rotational diffusion of TRPM6npWT was observed. Fluorescence lifetimes of the free TRPM6npWT and TRPM6npWT/CaM or TRPM6npWT/S100A1 complexes were measured and utilised to determine the quantum yield ratio (Q) of the bound to the free TRPM6npWT peptide. Subsequently, the fraction of bound TRPM6npWT peptide was plotted as a function of ligand concentration to define the equilibrium dissociation constants of TRPM6npWT/ligand complexes (Figure 1C–E). The dissociation constant (K_D_) values were determined to be 14.87 μM for TRPM6npWT/CaM and 17.80 μM for TRPM6npWT/S100A1. Our data indicate that TRPM6npWT/CaM-binding affinity is in the similar range as for the TRPM6npWT/S100A1 complex.

### 2.3. Characterisation of the TRPM6np/CaM Complex

**Figure 2:**
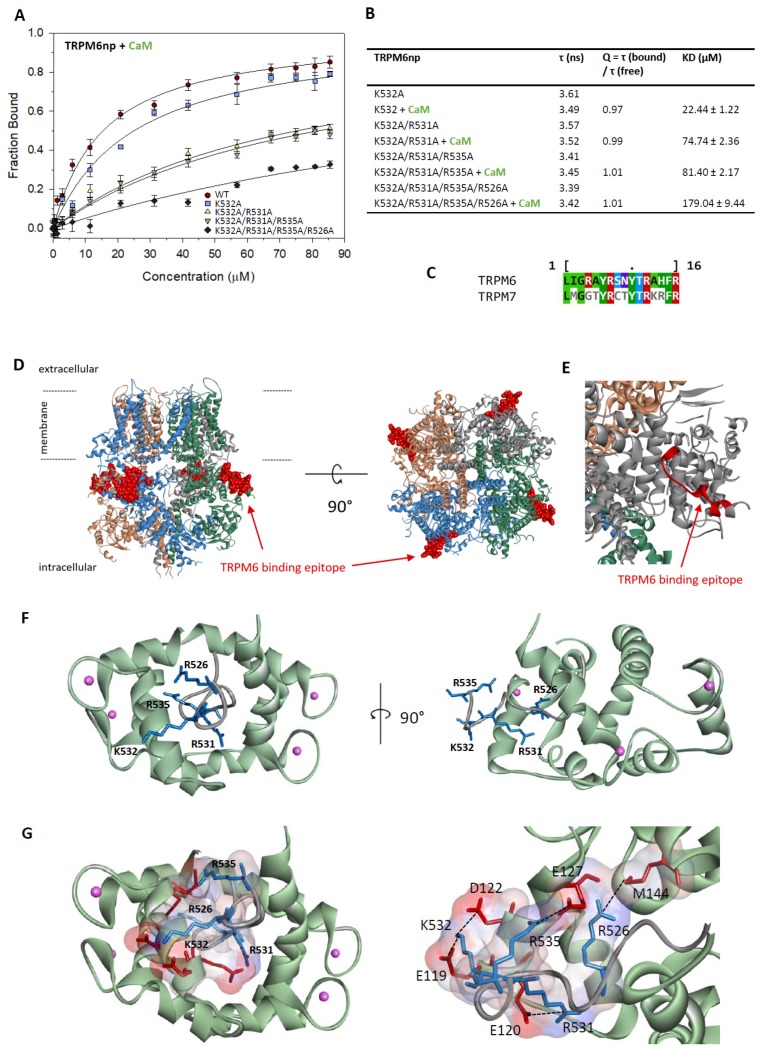
The TRPM6np/CaM complex characterisation: (**A**) The bound fractions of FITC-labelled TRPM6np as a function of CaM concentration obtained by steady-state fluorescence anisotropy measurement. The F_B_ of FITC-labelled TRPM6np (WT and K532A, K532A/R531A, K532A/R531A/R535A, and K532A/R531A/R535A/R526A mutants) calculated according to Equation (1) was plotted against particular CaM concentrations, and the best fit obtained using Equation (2) gave the binding isotherms (WT and mutants appropriate solid lines; see the Methods section). The error bars represent the standard deviation obtained from at least five measurements. (**B**) A summary of fluorescence lifetimes of FITC-labelled TRPM6np-derived mutants K532A, K532A/R531A, K532A/R531A/R535A, and K532A/R531A/R535A/R526A (free and bound); corresponding correction factors; and resultant equilibrium dissociation constants of the TRPM6np/CaM complex obtained by steady-state fluorescence anisotropy measurements. (**C**) The TRPM6np and its homology TRPM7 binding motif amino acid sequences alignment: The TRPM7 motif corresponds to a part of the CryoEM assigned structure (PDB: 5ZX5). (**D**) Schematic side and top views of TRPM6 in the membrane modelled according to homologous TRPM7 structural data (5ZX5): Each TRPM6 homomeric subunit is coloured differently. TRPM6np (red ball representation) displays the CaM and S100A1 binding domain locations in the whole TRPM6. (**E**) Detail of the TRPM6np binding domain location based on the TRPM7 channel homology model: The binding domain does not contain a secondary structure element. (**F**) Top and side view of TRPM6np/CaM complex representation: TRPM6np (backbone in grey) and CaM (backbone in light green) composing the binding interface. The basic residues (R526, R531, K532, and R535) of TRPM6np involved in the interactions are shown in blue. Calcium ions are shown in pink as scaled ball representations. (**G**) Full and detailed TRPM6np/CaM complex binding interface with surface representation of positively (R526, R531, K532, and R535 from TRPM6np, coloured in blue) and negatively (E119, E120, D122, E127, and M144 from CaM, coloured in red) charged residues involved in the non-covalent interactions. The colour convention has been used as in the Figure 2F representation.

The role of specific TRPM6np amino acid residues in the interactions with CaM was determined by alanine-scanning mutagenesis using a steady-state fluorescence anisotropy binding assay (Figure 2A,B). The alanine-scanning mutagenesis was performed at chosen basic residues of TRPM6np because only these amino acid residues were predicted to ensure a specific TRPM6np interaction with the ligands. We have designed the alanine-replacement mutants of single (K532A), double (K532A/R531A), triple (K532A/R531A/R535A), and tetra (K532A/R531A/R535A/R526A) basic amino acids in the TRPM6npWT peptide.

The fluorescence anisotropy data revealed only a small decrease in CaM-binding affinity of a single mutant (K532A, K_D_ = 24.44 µM) in comparison to TRPM6npWT (14.87 μM). Additional mutation of the neighbouring arginine residue generated a double-mutated peptide (K532A/R531A) with significantly lower binding to CaM (K_D_ = 74.74 µM). These results indicate the importance of tandem basic residues (K532A/R531A) for strong TRPM6npWT/CaM binding. In contrast to R531A substitution, the additional mutation of arginine residue (triple-mutated peptide (K532A/R531A/R535A)) did not cause a significant decrease of CaM binding (K_D_ = 81.40 µM) compared to double mutant K532A/R531A. The affinity of TRPM6np to CaM was reduced in tetra-mutated peptide (K532A/R531A/R535A/R526A), where the additional substitution of R526A caused an increase of K_D_ to 179.04 µM. Our results have confirmed the specificity of TRPM6np/CaM complex formation due to non-covalent interactions.

### 2.4. Characterisation of the TRPM6np/S100A1 Complex

**Figure 3:**
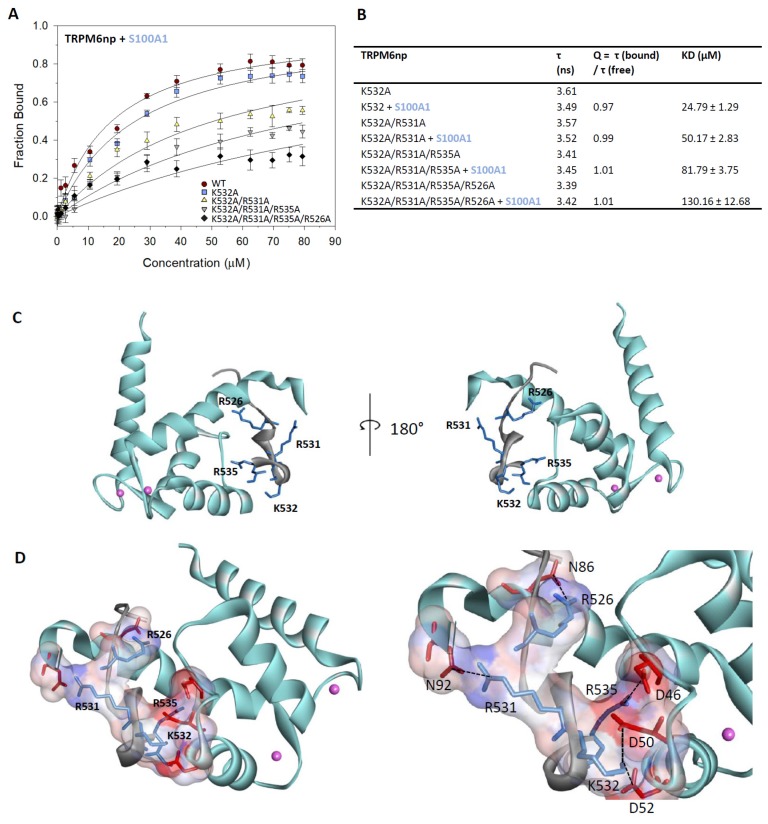
The TRPM6np/S100A1 complex characterisation: (**A**) The bound fractions of FITC-labelled TRPM6np as a function of S100A1 concentration obtained by steady-state fluorescence anisotropy measurement. The F_B_ of FITC-labelled TRPM6np (WT and K532A, K532A/R531A, K532A/R531A/R535A, and K532A/R531A/R535A/R526A mutants) calculated according to Equation (1) was plotted against particular S100A1 concentrations, and the best fit obtained using Equation (2) gave the binding isotherms (WT and mutants appropriate solid lines; see the Methods section). The error bars represent the standard deviation obtained from at least five measurements. (**B**) A summary of fluorescence lifetimes of FITC-labelled TRPM6np-derived mutants K532A, K532A/R531A, K532A/R531A/R535A, and K532A/R531A/R535A/R526A (free and bound); corresponding correction factors; and resultant equilibrium dissociation constants of the TRPM6np/S100A1 complex obtained by steady-state fluorescence anisotropy measurements. (**C**) Top and side view of TRPM6np/S100A1 complex representation: TRPM6np (backbone in grey) and S100A1 (backbone in light blue) composing the binding interface. The basic residues (R526, R531, K532, and R535) of TRPM6np involved in the interactions are shown in blue. Calcium ions are shown in pink as scaled balls representations. (**D**) Full and detailed TRPM6np/ S100A1 complex binding interface with surface representation of positively (R526, R531, K532, and R535 from TRPM6np, coloured in blue) and negatively (D46, D50, D52, N86, and N92 from S100A1, coloured in red) charged residues involved in the non-covalent interactions: The colour convention has been used as in the Figure 3C representation.

To assess the impact of TRPM6np basic residues on the interaction with S100A1, we have performed steady-state fluorescence anisotropy measurements using a set of previously designed (the same mutants as used for binding assay with CaM.; see the section above) alanine-replacement mutants in the TRPM6np region (Figure 3A,B). The single- (K532A), double- (K532A/R531A), triple- (K532A/R531A/R535A), and tetra- (K532A/R531A/R535A/R526A) mutated peptides were used for the measurements.

The K_D_ value for the complex of single-mutated peptide (K532A) with S100A1 remained similar (K_D_ = 24.79 µM) to that for TRPM6npWT/S100A1 (K_D_ = 17.80 μM). A decrease of TRPM6npWT binding affinity to S100A1 was achieved by additional substitution of R531 to alanine (K532A/R531A, K_D_ = 50.17 μM). Our data indicate the significance of this residue in TRPM6npWT/S100A1 complex formation, but its contribution to binding is not as significant as in the case of the TRPM6npWT/CaM complex, where K532A/R531A mutations led to a more than 2-fold decrease of binding affinity. The placement of three simultaneous mutations (K532A/R531A/R535A) in the TRPM6np region caused further reduction of binding affinity to S100A1 (K_D_ = 81.79 μM), and finally, in tetra-mutated peptide (K532A/R531A/R535A/R526A), the K_D_ value increased to 130.16 μM. These data confirmed the pivotal role of predicted basic amino acid residues in both complexes. It can be concluded that the basic TRPM6np residues involved in the CaM interactions are also involved in S100A1 interactions.

### 2.5. TRPM6np Molecular Modelling and CaM and S100A1 Docking

Homology modelling methods were used to build a molecular model of TRPM6, taking into account that the structure of the TRPM6 channel is still unknown. The structure of the mouse TRPM7 channel (PDB: 5ZX5) [22] with a 61.74% sequence identity to TRPM6 (Figure S1) was used as a template to build the TRPM6 model by SWISS-MODEL [50]. Specifically, according to the sequence of TRPM6np, the homology region of TRPM7 was selected (UniProt: Q96QT4.1; sequence: LMGGTYRCTYTRKRFRL) (Figure 2C) [51]. The high sequence-homology degree of the TRPM6 and TRPM7 regions made it possible to select the TRPM6 model built by the SWISS-MODEL. The TRPM6np structure from this homology model was separated and optimised by energy minimisation using Molecular Operating Environment software [52].

TRPM6np was consequently docked into CaM and S100A1 by the ClusPro2.0 method and server [53] (see the Methods section, Section 4.6). The TRPM6np/CaM complex (Figure 2F,G) was adjusted according to its similarity with the CaM binding domain of TRPV1 containing 1–10 hydrophobic motifs. The TRPV1p/CaM complex was solved by X-ray crystallography (PDB: 3SUI) [48]. The TRPM6np/S100A1 complex (Figure 3C,D) was adjusted based on its similarity with the crystal structure of the complex RyR1P12 peptide with S100A1 (PDB: 2K2F) [45]. The TRPM6np/CaM and TRPM6np/S100A1 complexes were compared with structural information derived from the analysis of TRPV1/CaM and RyR1P12/S100A1 complexes [45,48]. The hydrophobic motifs present in three different positions of TRPM6np (Y525-F534 (1–10 motifs), L520-A524-Y529 (1–5–10 motifs), and I521-F534 (1–14 motifs) indicate strong hydrophobic interactions with CaM and S100A1. Ligand docking has confirmed that R526, R531, K532, and R535 of TRPM6np are crucial for the interactions with negatively charged residues of CaM and S100A1 (E119, E120, D122, E127, and Met144 in CaM and D46, D50, D52, N86, and N92 in S100A1; Figure 2G and Figure 3D). The molecular modelling of TRPM6np/CaM and TRPM6np/S100A1 binding interfaces thus supports data from the binding assay.

## 3. Discussion

We decided to investigate the binding of two known CBPs, CaM and S100A1, to the TRPM6 N-terminus. CaM is a very well-known modulator of the activity of many receptors as well as for TRPs [37,41,54]. Actually, the detailed structural analysis of CaM in the complex with the TRPV6 receptor leading to channel inhibition has been described recently [42]. The modulation of receptors by S100A1 is also well described, e.g., for RyR1 [55], the authors described that RyR1 shared the CaM and S100A1 binding sites and the activating effect of these bindings on the channel function. Therefore, we decided to investigate if CaM and S100A1 can also share the binding site at TRPM6np. The TRPM6np capability of separately binding CaM and S100A1 by has been confirmed by steady-state fluorescence anisotropy. The binding affinity is almost the same for both of the ligands, and the obtained K_D_ values are in a range of micromolar concentrations and is around 5–10 times lower than for the K_D_ values of other in vitro investigated complexes of TRP channels (TRPM1, TRPM3, TRPM4, TRPC6, and TRPV1) with CaM and/or S100A1 [29,30,31,47,56]. The strong binding affinity of the complex is dependent on complementarity of interacting amino acids in the binding site. Molecular modelling predicted potential binding interfaces with typical CaM and S100A1 binding characteristics [45,48]. The formation of these complexes is a sequential process where CaM or S100A1 nonspecifically interacts with hydrophobic residues of the TRP binding domain. Based on this TRP adaptation, basic amino acids become more accessible for the interaction with negative amino acids of CaM or S100A1 [30,31]. Clusters of mutated basic amino acids K532A/R531A/R535A/R526A in specific peptide analogues have been showed a substantial decrease of CaM or S100A1-binding affinities and has determined participation of these residues on the interactions. The different binding affinities of the mutants have indicated that the structural parameters of ligand binding are different, which has been supported by results of molecular modelling. The fifth R523 N-terminal residue of TRPM6np certainly contributes to the positive character of the basic amino acid cluster, which is important for binding to TRPM6np. The molecular models of TRPM6np/CaM and TRPM6np/S100A1 which were developed confirmed that R526A, R531A, K532A, and R535A form salt bridges directly with negatively charged amino acids CaM or S100A. The R523 is not involved in such strong interactions as apparent at the molecular models; therefore, we primarily targeted electrostatic compatibility at the binding site that includes four residues R526A, R531A, K532A, and R535A. Mutual competitions of CaM and S100A1 for overlapping binding sites of TRPs on N- and C-termini and RyR1 have already been characterised many times previously [30,45,55,56,57]; therefore, we anticipate mutual competition of CaM and S100A1 for TRPM6np as well. We have not investigated the specific role of the hydrophobic residues in the TRPM6np binding domain because the hydrophobic patch of the receptor mostly forms nonspecific interactions with the ligands. The main specificity of the complex is always led through basic residues of the receptor and acidic residues of CaM or S100A1 [29,31,42,47,56]. The in vitro Ca^2+^ dependency interactions of the TRP channel protein/peptide segments (specifically: TRPM3, TRPC6, and TRPV1) with CaM and/or S100A1 has been validated in vitro by spectroscopy methods in our previous experiments [30,47,56], which allowed us now the use of experimental buffers with the same Ca^2+^ concentrations (2 mM). Therefore, it was supposed that the CaM/TRPM6np and S100A1/TRPM6np complexes were formed by ligands with bound Ca^2+^. Since the modulation activity of CaM and S100A1 at TRPM6 is still not known, this should be investigated by electrophysiology measurements.

The molecular modelling of the TRPM6np/CaM and TRPM6np/S100A1 complexes was performed to visualise the specific character of the binding interfaces for both complexes. High sequence similarity of TRPM6 with TRPM7 made it possible to use homology modelling for this purpose [22]. We have identified the TRPM7 region in the Cryo-EM structure that corresponds to the TRPM6np sequence and that exhibits a structural form with no secondary structure element. Given the character of this region, we can assume that TRPM6np can behave in a rather adaptive way, depending on its binding partner. The binding interface of TRPM6np/CaM corresponds to previously published structures of receptor-fragment/CaM complexes [44,48,54,58]. As we published earlier, the amino acids of TRP in the CaM-binding domain cooperate as a cluster via ligand interaction [30,31,47]. The binding role of CaM amino acids at the position around 130 has been confirmed in the TRPV6/CaM and TRPV5/CaM structures, and the amino acids have been identified as critical positions for the interactions. In the presented molecular model, we have confirmed the same CaM amino acids involved in interactions with TRPM6np (specifically: E120 and M144) [36]. The interpretation of the TRPM6np/CaM and TRPM6np/S100A1 models is strongly supported by data from fluorescence anisotropy experiments, indicating the synergy of the basic residues in individual clusters. The TRPV6/CaM and TRPV5/CaM structures confirmed the presence of mutual binding sites for one CaM molecule by different TRP tails or subunits [36,42]. This can drive a coordinated conformational changes ongoing across the whole channel, leading to its functional modulation [36,59]. This complex process can be used to multiply the ligand signal into a channel structure to transfer structural changes more rapidly to the target location of the channel via its allostery. The ligand multi-binding character of TRPs has been discussed in previous publications [29,60].

The TRPM6 N-terminus is a universal binding domain for CaM and S100A1 in the presence of Ca^2+^. The binding affinities of the TRPM6np/CaM-Ca^2+^ and TRPM6np/S100A1-Ca^2+^ complexes have been investigated in vitro by the fluorescence spectroscopy method, which has revealed the micromolar range of the interactions. Homology modelling using the TRPM7 Cryo-EM structure as a template has provided TRPM6np structurally adaptive character through the binding process. Intracellular parts of all TRPs have a common feature—their mostly disordered character, which allows their binding domains to be implicated in a wide range of modulatory molecules. Although TRPM6 has been studied quite well, information about its N-terminal function is not yet well understood. This study provides a new in vitro insight at the role of CBP as potential TRPM6 modulators and reveals new information on the channel signalling pathways. While no functional TRPM6 data (i.e., physiological consequences) is presented, the interactions with CaM and S100A1 are likely to provide stimulatory follow-up studies in this field. Therefore, the discovered new TRPM6 binding domains and interaction plasticity information could serve to develop new drugs.

## 4. Methods

### 4.1. TRPM6-Binding Domain Design and Synthesis

The DNA sequence of human TRPM6 has been searched for a potential CaM and S100A1-binding motifs using the Calmodulin Target Database [49]. To verify a new CaM- and S100A1-binding domain at TRPM6, we have selected a potential binding domain from the N-terminus TRPM6. The selected binding domain is referred to here as TRPM6np (UniProt: Q9BX84–2; sequence: 520-LIGRAYRSNYTRKHFR-535). TRPM6np wild type (TRPM6npWT) and its alanine scan analogues have been synthesised into the variants: K532A, R531A/K532A, R526A/R531A/K532A, and R526A/R531A/K532A/R535A as peptides (GenicBio Limited, Shanghai, China). All of these peptides were N-terminally labelled with fluorescein-5-isothiocyanate (FITC). The peptide probes were dissolved in a 50 mM Tris-HCl buffer (pH 7.5) containing 500 mM NaCl and 1 mM CaCl_2_.

### 4.2. Expression and Purification of CaM and S100A1

CaM and S100A1 were expressed from pET3a and pET28b expression vectors in *E. coli* BL21 cells, respectively. The cultures were incubated at 37 °C in Lysogeny Broth medium with ampicillin or kanamycin until the OD_600_ reached 0.9. The cell suspension was cooled at 25 °C, and protein expression was induced by 0.5 mM IPTG for 12 h. The cells were resuspended in 50 mM Tris-HCl buffer (pH 7.5) containing 2 mM ethylenediaminetetraacetic (EDTA), 2 mM β-mercaptoethanol, and 0.2 mM phenylmethylsulphonyl fluoride (PMSF) and disrupted by sonication, and 5 mM CaCl_2_ was added to the supernatant. CBPs were purified using affinity chromatography on Phenyl Sepharose CL-4B (GE Healthcare, Brondby, Denmark), where 50 mM Tris-HCl buffer (pH 7.5) containing 100 mM NaCl and 1.5 mM EDTA was used for elution. Size-exclusion chromatography on a Superdex 75 10/300 GL column (GE Healthcare, Brondby, Denmark) was used as the final purification step. The proteins were eluted by 50 mM Tris-HCl buffer (pH 7.5) containing 500 mM NaCl and 1 mM CaCl_2_, and their purity was verified by SDS-PAGE. The identity of proteins was confirmed by mass spectrometry.

### 4.3. Steady-State Fluorescence Anisotropy Measurements

Steady-state fluorescence anisotropy experiments were performed at room temperature on a photon counting spectrometer PC1 (ISS Inc., Champaign, Illinois, USA). TRPM6npWT and its mutants were dissolved in a buffer containing 50 mM Tris-HCl (pH 7.5), 150 mM NaCl, and 2 mM CaCl_2_ for a final concentration of 1 μM. The samples were titrated in a 2-mm path cuvette with increasing aliquots of 100 μM CaM or S100A1. Fluorescence was excited at 495 nm; the intensity of emission in parallel (I_II_) and perpendicular (I_﬩_) orientations to the direction of the polarised excitation was obtained at 520 nm by switching the emission polariser. The steady-state fluorescence anisotropy value (r) was calculated from the equation r = (I_II_ − I_﬩_) / (I_II_ + 2I_﬩_). Further analysis was based on the mean anisotropy value acquired from five independent measurements at each CaM or S100A1 addition. The fractions of bound CaM or S100A1 were given by fraction bound (F_B_)

F_B_ = (r_obs_ − r_min_)/[(r_max_ − r_obs_) Q + (r_obs_ − r_min_)] (1)

where r_max_ is the anisotropy of a saturated binding complex, r_min_ stands for the anisotropy of a peptide probe without a ligand, and r_obs_ is the anisotropy at a particular CaM or S100A1 concentration. Q represents the quantum yield ratio of the bound to the free peptide, calculated from fluorescence lifetimes (τ) according to equation Q = τ_bound_/τ_free_.

To determine the equilibrium dissociation constant (K_D_), F_B_ was plotted as a function of CaM or S100A1 concentration and fitted by as follows [24]:

(2) $$F_{B}=\frac{K_{D}+\left[P1\right]+\left[P2\right]-\sqrt{{\left(K_{D}+\left[P1\right]+\left[P2\right]\right)}^{2}-4\left[P1\right]\left[P2\right]}}{2\left[P1\right]},$$

where (*P*1) is the TRPM4np concentration and (*P*2) is the CaM or S100A1 concentration. Nonlinear data fitting was performed using SigmaPlot 11.0 (Systat software Inc., San Jose, CA, USA).

### 4.4. Lifetime Experiments

Lifetimes were evaluated at room temperature in a drop placed on a coverslip and inserted into an inverted confocal microscope IX83 (Olympus, Tokyo, Japan) equipped with time-correlated single photon counting electronics and cooled GaAsP hybrid detectors (all PicoQuant, Berlin, Germany). TRPM4np fluorescence was excited at 485 nm by an LDH-485 picosecond laser (PicoQuant, Berlin, Germany). Emission decays were collected in the epi-fluorescence mode using a combination of a 488-nm dichroic long-pass filter (Olympus, Tokyo, Japan) and a 520/35 bandpass filter (Semrock, Rochester, NY, USA) in the detection path. The intensity-weighted mean fluorescence lifetimes used in the calculation of the Q correction factor were evaluated as follows:

(3) $$\tau_{\mathrm{mean}}=\sum_{i}\alpha_{i}\tau_{i}^{2}/\sum_{i}\alpha_{i}\tau_{i},$$

where *τ_i_* stands for fluorescence lifetimes and *α_i_* are the corresponding amplitudes.

### 4.5. Model Building

The TRPM6np (Uniprot: Q9BX84–2) molecular model was generated via homology modelling methods using SWISS-MODEL [49]. The structure of the mouse TRPM7 channel (PDB: 5ZX5) [22] with the highest sequence similarity to TRPM6 has been used as a template to build the TRPM6np molecular model. The TRPM6np models were built, and one representative model was carefully chosen based on the positions of the basic and hydrophobic residues to be exposed to the solvent. Additional criteria were applied on the geometric parameters of the potential CaM- and S100A1-binding sites. The TRPM6np molecular model was then optimised by energy minimisation using MOE software [51] and checked for errors in the three-dimensional protein structure. The quality of the resulting structure was further assessed using STING Millenium [61] and ProSA-web [62].

### 4.6. Ligand Docking

The docking of CaM and S100A1 into the TRPM6np homology model was performed using the ClusPro program [53,63,64]. CaM and S100A1 templates were selected from the structures of complexes TRPV1 with CaM/Ca^2+^ (PDB: 3SUI) and RyR1 with S100A1/Ca^2+^ (PDB: 2K2F). The molecular models of both TRPM6np/CaM and TRPM6np/S100A1 were optimised by energy minimisation using MOE software [53] and checked to identify errors in the three-dimensional protein structure. The schematic representations of the TRPM6npWT/CaM and TRPM6npWT/S100A1 complexes were generated using Discovery Studio Visualizer [65]

## Supplementary Materials

Supplementary materials can be found at https://www.mdpi.com/1422-0067/20/18/4430/s1.

## Abbreviations

ATP	adenosine trisphosphate
CaM	calmodulin
CBPs	calcium-binding proteins
Cryo-EM	Cryogenic Electron Microscopy
IDPs	intrinsically disordered proteins
PIP2	phosphatidylinositol 4, 5- bisphosphate
RyR	rhyanodine receptor
S100A1	S100 calcium-binding protein A1
TRP	transient receptor potential channel
TRPM	transient receptor potential channel melastatin
TRPM6np	transient receptor potential channel melastatin 6 N-terminal peptide
TRPs	transient receptor potential channels
TRPV	transient receptor potential channels
WT	wild type
