# Supplementary material for: TRPM6 N-Terminal CaM- and S100A1-Binding Domains
Source: Int J Mol Sci. 2019 Sep 9;20(18):4430. doi: 10.3390/ijms20184430 (PMC6770577; doi:10.3390/ijms20184430)
Supplement: Supplementary file 1 [file ijms-20-04430-s001.pdf]

SUPPLEMENTS

A

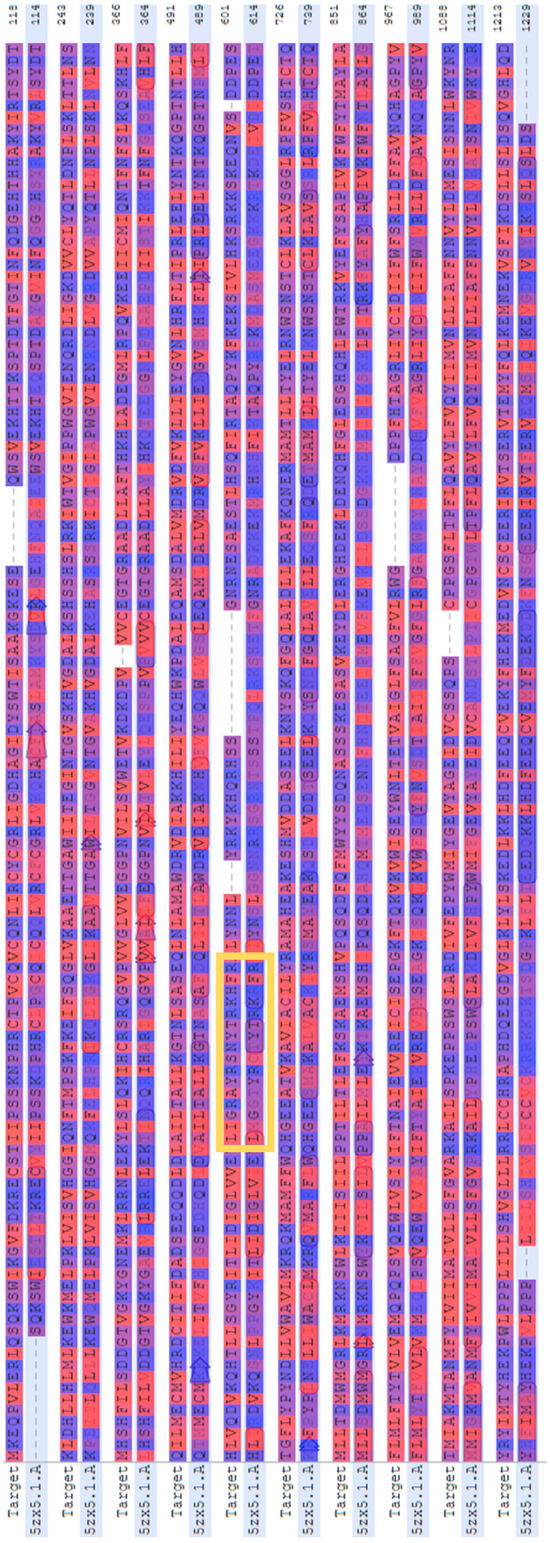

B

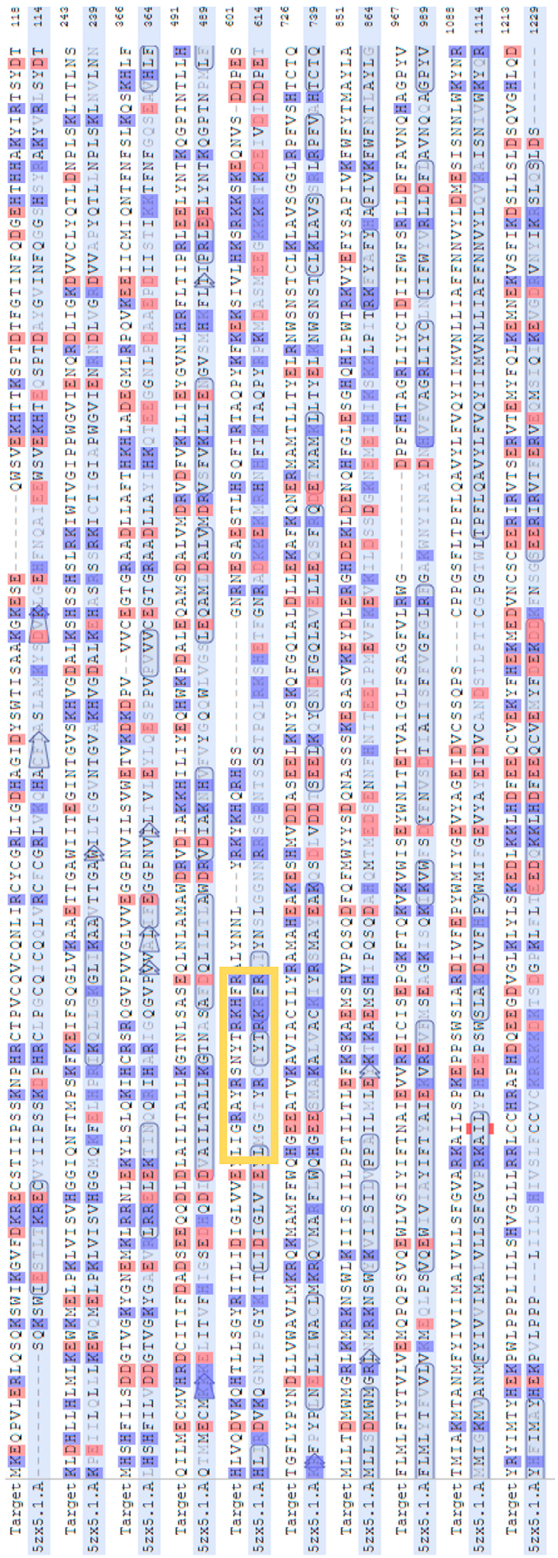

S1. Alignment of TRPM6 with TRPM7. The TRPM6 and TRPM7 amino acid sequences alignment. The alignment was performed according to TRPM7 part which has been assigned to structure by CryoEM. (A) The hydrophobic and (B) the polar character of TRPM6/TRPM7 alignments. Specific TRPM6np binding epitope aligned with TRPM7 region is labelled in yellow frame. TRPM6 sequence corresponds to human; TRPM7 sequence corresponds to part of CryoEM assigned structure (PDB: 5ZX5).
